# Inhibition of p53 inhibitors: progress, challenges and perspectives

**DOI:** 10.1093/jmcb/mjz075

**Published:** 2019-08-20

**Authors:** Gema Sanz, Madhurendra Singh, Sylvain Peuget, Galina Selivanova

**Affiliations:** Department of Microbiology, Tumor and Cell Biology, Biomedicum 8C, Karolinska Institute, Sweden

**Keywords:** p53, tumor suppression, anti-cancer therapy, targeted drugs, transcription factor, immune response

## Abstract

p53 is the major tumor suppressor and the most frequently inactivated gene in cancer. p53 could be disabled either by mutations or by upstream negative regulators, including, but not limited to MDM2 and MDMX. p53 activity is required for the prevention as well as for the eradication of cancers. Restoration of p53 activity in mouse models leads to the suppression of established tumors of different origin. These findings provide a strong support to the anti-cancer strategy aimed for p53 reactivation. In this review, we summarize recent progress in the development of small molecules, which restore the tumor suppressor function of wild-type p53 and discuss their clinical advance. We discuss different aspects of p53-mediated response, which contribute to suppression of tumors, including non-canonical p53 activities, such as regulation of immune response. While targeting p53 inhibitors is a very promising approach, there are certain limitations and concerns that the intensive research and clinical evaluation of compounds will hopefully help to overcome.

## Introduction

Since p53 discovery 40 years ago, it has been established that p53 is a transcriptional factor, which binds to the promoters of its target genes in a sequence-specific manner and regulates their expression, thereby controlling cell cycle and cell death. In unstressed cells, p53 has low activity; however, upon its activation by oncogenes, DNA damage, and other types of stresses, p53 blocks the proliferation of pre-malignant and malignant cells or eliminates them by inducing apoptosis (reviewed in Vogelstein, et al., 2000; Vousden and Prives, 2009; Kastenhuber and Lowe, 2017). In addition to the canonical functions, such as induction of growth arrest, senescence, apoptosis and facilitation of DNA repair, non-canonical p53 activities, including anti-oxidant response, ferroptosis, regulation of metabolism and autophagy, modulation of tumor stroma and immune respons, as well as the block of invasion and metastasis, greatly contribute to anti-cancer properties of p53 (Vousden and Prives, 2009; Kastenhuber and Lowe, 2017).

Early studies provided an ample evidence for p53 being *bona fide* tumor suppressor by demonstrating a 100% cancer penetrance in different strains of mice lacking p53. *TP53* germline mutation is associated with Li–Fraumeni syndrome, which is characterized by an increased risk of cancers in tissues of different developmental origin (Bougeard et al., 2015). New generation sequencing of thousands of cancer genomes has confirmed that p53 mutations is the most frequent genetic alteration in cancer (Sjöblom et al., 2006; Lawrence et al., 2013). On the other hand, in tumors carrying wild-type p53, its function is abolished by its inhibitors, such as MDM2 and MDMX (Vogelstein et al., 2000; Vousden and Prives, 2009).

Studies taking advantage of mouse strains expressing ‘switchable’ p53 genes have uncovered a crucial role of p53 reconstitution in regression of already established tumors—lymphomas, soft tissue sarcomas, and hepatocellular carcinomas (Martins et al., 2006; Ventura et al., 2007; Xue et al., 2007). Importantly, these studies emphasized the absence of growth suppression in normal tissues upon p53 re-establishment by genetic means (Christophorou et al., 2005).

## p53 pathway as a target for anti-cancer drug development

Detailed molecular analysis and next-generation sequencing (NGS) of hundreds of human cancers revealed an almost indefinite number of combinations of mutations, chromosomal aberrations, copy number changes, and epigenetic alterations. However, these very diverse cancer lesions converge on a few key pathways.

To successfully fight cancer, we need to focus on these most crucial pathways and find the best targets within these. To identify the best targets, we should apply the following criteria: the factor is a critical player in essential pathways affected in many cancers; it is non-redundant and is involved in different aspects of tumor development; targeting this factor results in elimination of tumor cells, but does not kill normal cells.

p53 fits these criteria very well; inactivation of the p53 tumor suppressor function is required for the development and maintenance of most human cancers. Importantly, p53 is negatively controlling most of the hallmarks of cancer: deregulated proliferation and cell death, replicative immortality, angiogenesis, invasion and metastasis, metabolism and genomic instability, as well as immune response (Kastenhuber and Lowe, 2017). p53 is a non-redundant core signalling molecule; although p53 family members p73 and p63 share the DNA binding specificity among them, only p53 has a crucial role in preventing cancer development. This is supported by the results of NGS showing that p53 is the most frequently mutated gene in cancer, inactivated by mutations in the majority of cancer types. In tumors maintaining wild-type p53, its tumor suppressor function is compromised by the expression of a numerous negative regulators. And, finally, p53 reconstitution triggers apoptosis in many types of cancer cells, while its effects in most normal tissues appear to be minimal. Thus, p53 is a perfect target for cancer therapy.

**Figure 1:**
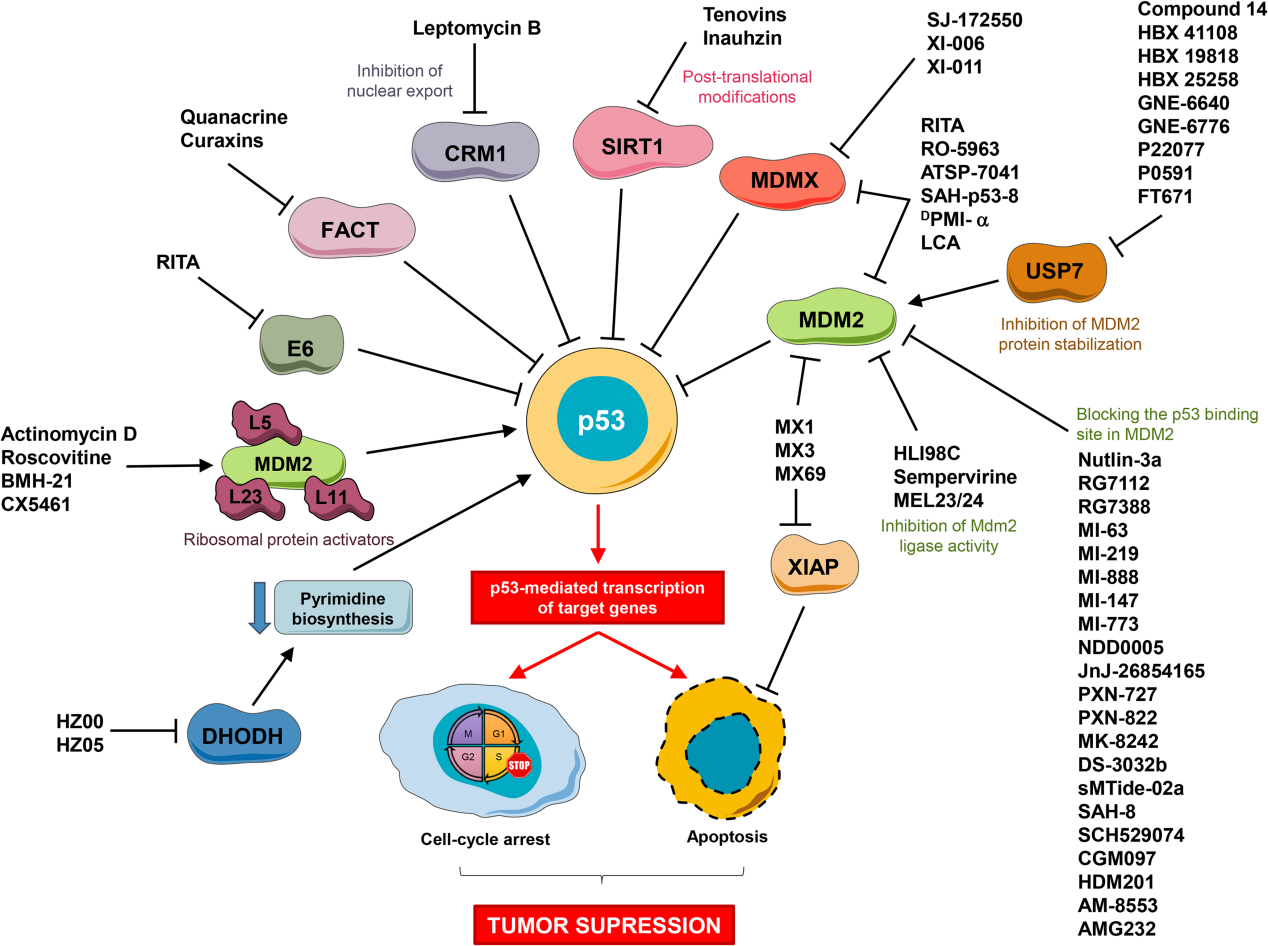
Major approaches for pharmacological reactivation of p53.

Due to the unique mode of p53 inactivation in cancer, restoration of p53 appears to be feasible. In contrast to other tumor suppressors such as Rb, p16, or PTEN, the p53 protein is usually expressed in tumors, although its function is ablated. However, the fact that p53 is a transcriptional factor have made the idea of p53 reactivation unpopular in the past, since transcriptional factors were deemed ‘undruggable’ until very recently. Latest advances have proven these views wrong and made it possible to develop different strategies for the restoration of p53 activity, depending on the type of p53 inactivation. Reactivation of mutant p53 protein by stabilizing its folding with small molecules appears to be a promising strategy, i.e. development of small molecule PRIMA-1MET/APR246 (Bykov et al., 2002), which is now being evaluated in a number of clinical trials, including Phase III trials. In wild-type p53 tumors, the major approach is to block p53 inhibitors, the major focus being on MDM2 and MDMX (Figure 1).

## Restoration of wild-type p53 function

In the absence of p53 mutations in tumors, p53 function is frequently impaired due to different alterations which result in the enhanced activity of its two major negative regulators, MDM2 and its homologue MDMX (encoded by *MDM4* gene). MDM2 regulates p53 via different mechanisms. Due to its E3 ligase activity, MDM2 can either monoubiquitinate p53, promoting its nuclear export, or polyubiquitinate, inducing p53 degradation by the proteasome (Li and Lozano, 2013). In addition, MDM2 can bind to the N-terminal transactivation domain of p53, blocking its transcriptional activity. While MDMX does not have E3 ligase activity, it inhibits p53 by binding to its N-terminal transactivation domain, similar to MDM2. The formation of homo-oligomers through the RING finger domains is necessary for the ubiquitin ligase function of MDM2. MDMX cannot form such homo-oligomers, but hetero-oligomerization of MDM2 and MDMX renders a more efficient E3 ligase activity (Francoz et al., 2006; Wade et al., 2013). Mice lacking either of these two p53 inhibitors are not viable. They die *in utero* due to growth arrest or apoptosis in embryonic tissues, while in the p53-null background, deletion of MDM2 or MDM4 is not affecting viability of mice (reviewed in Li and Lozano 2013; Khoo et al. 2014; Moyer et al. 2017). Thus, MDM2 and MDMX (MDM4) have non-redundant functions and are both critical regulators of p53-induced growth suppression.

Multiple mechanisms of enhanced activity of these two inhibitors have been observed: amplification of the genes encoding MDM2 or MDMX, enhanced transcription or translation, altered posttranslational modifications, overexpression of positive regulators (Twist, WIP1, Akt), or the absence of negative regulators (p14ARF, ATM, CHK2) (Selivanova, 2014). A single-nucleotide polymorphism at position 309 (SNP309) in MDM2 promoter generates a binding site for the transcription factor SP1, increases MDM2 expression, and leads to mitigated p53 activity and acceleration of tumor development in humans (Bond et al., 2004). All these alterations converge on p53/MDM2/MDMX interaction leading to impaired p53 activity even in the absence of p53 mutations. Thus, blocking the MDM2/p53 interaction to reactivate the wild-type p53 function is an attractive therapeutic strategy for cancer treatment. A number of small-molecule inhibitors of MDM2/X have been developed up to date (Wade et al., 2013; Selivanova, 2014). These compounds target MDM2 and/or MDMX, p53, or upstream regulators (Figure 1).

### MDM2 inhibitors

Small molecules targeting MDM2, which block the p53 binding site of MDM2 and prevent its interaction with p53 via steric hindrance, include nutlins (Vassilev et al., 2004), spirooxindoles (Ding et al., 2006), benzodiazepinediones (Grasberger et al., 2005), and piperidinones (Sun et al., 2014). Nutlin3a stabilizes and activates p53 by binding to the hydrophobic pocket of MDM2, thus preventing p53 to engage in complex with MDM2, leading to p53 stabilization and activation. This results in expression of p53 downstream targets such as p21 or PUMA and the induction of cell cycle arrest or apoptosis (Vassilev et al., 2004). The nutlin analogue RG7112 was the first MDM2 inhibitor tested in clinic in liposarcoma patients with MDM2 amplification (Vu et al., 2013). The most recent derivative of nutlin, RG7388, showed improved affinity and potency *in vitro* and *in vivo* (Ding et al., 2013).

Spirooxindole-containing compounds (MI series) are another class of high-affinity compounds-antagonists of MDM2, designed specifically to mimic the three key hydrophobic residues (Phe19, Trp23, and Leu26) of p53, making contacts within the MDM2 pocket. MI-63 and MI-147 induce cell growth arrest in several cell lines due to p53 activation (Ding et al., 2006; Yu et al., 2009). Moreover, MI-147 suppressed tumor growth *in vivo* in SJSA-1 xenograft model, either alone or in combination with irinotecan (Yu et al., 2009), while MI-888 showed anti-tumor activity without evident toxicity upon oral administration (Zhao et al., 2013). MI-773 (SAR405838) significantly decreased the tumorigenecity of de-differentiated liposarcoma xenografts with high levels of MDM2 (Bill et al., 2016). However, acquisition of p53 mutations confers resistance to MDM2 inhibitors (Jung et al., 2016).

*De novo* design, based on the binding mode of previously known MDM2 inhibitors, resulted in the development of a novel scaffold for inhibiting MDM2. Piperidinones AM-8553 (Rew et al., 2012) and AMG232 are another type of selective piperidinone inhibitor of MDM2–p53 interaction, which also showed anti-tumor effect in SJSA-1 osteosarcoma xenograft mouse model. AMG232 is currently being tested in Phase I study in patients with different types of solid and hematological tumors (Sun et al., 2014).

Design of ‘stapled’ p53-based peptides is another promising approach to prevent the p53–MDM2 interaction. Peptides are stapled by the addition of a hydrocarbon linkage that stabilizes the α-helical structure, confers resistance to proteases, and promotes cellular uptake (Schafmeister et al., 2000; Walensky et al., 2004; Bird et al., 2010). The first synthetized stapled peptide SAH-p53-8 (stabilized α-helix of p53) induced apoptosis in osteosarcoma SJSA-1 cells overexpressing MDM2 by reactivating the p53 signalling pathway (Bernal et al., 2007). However, quite high concentrations of SAH-p53-8 are required to prevent p53/MDM2 complex formation. sMTide analogues were identified by phage display techniques and further optimized to get a higher and improved binding affinity to the p53 binding cleft in MDM2. Phage-derived analogues, sMTide-02/02A, induce G1/G2 arrest in cells harboring wild-type p53, making them useful for cyclotherapy (Brown et al., 2013).

Inhibition of the E3 ligase activity of MDM2 is an alternative strategy to block it. Several small molecules have been identified for this purpose. HLI98 small-molecule family (Yang et al., 2005) and sempervirine (Sasiela et al., 2008) obstruct the MDM2 ligase activity, while MEL23 and MEL24 block the E3 ligase activity of the Mdm2/MdmX hetero-complex (Herman et al., 2011).

### MDMX inhibitors

Although some MDM2 inhibitors can also bind to MDMX (Figure 1), the structural differences in the p53-binding pocket between these two proteins lead to low affinity of MDM2 inhibitors to MDMX. This makes them inefficient in tumors with deregulated MDMX (Wade et al., 2013). MDMX is often overexpressed in different cancer types, for example, in melanoma (Marine, 2011). Therefore, development of specific MDMX inhibitors and/or dual MDM2/MDMX inhibitors is desirable. The first small-molecule MDMX inhibitor was SJ-172550. By displacing p53 from its binding pocket in MDMX, it induced apoptosis in retinoblastoma cells expressing high levels of MDMX and had an additive effect in combination with MDM2 inhibitor nutlin3a (Reed et al., 2010). XI-011 and XI-006, identified by MDMX promoter-linked luciferase assay, impede the MDMX promoter activity, leading to apoptosis in MCF7 cells and showed an additive effect in combination with nutlin3a (Wang et al., 2011).

### Dual inhibitors targeting MDM2 and MDMX

As mentioned above, dual targeting of MDM2/MDMX might be required to get a complete reactivation of p53 in tumors with overexpressed MDMX. Small molecule RO-5963 blocks homo- and heterodimerization of MDM2 and MDMX, triggering the activation of p53 signalling pathway and the induction of apoptosis (Graves et al., 2012). Small molecule RITA (i.e. reactivation of p53 and induction of tumor cells’ apoptosis) was previously identified by us using a phenotypic screen of the National Cancer Institute (NCI) library. RITA displayed efficient induction of apoptosis by inhibiting MDM2 and MDMX in cancer cells as well as in mouse xenografts (Issaeva et al., 2004; Enge et al., 2009; Spinnler et al., 2011). The exact mechanism of RITA action remains to be elucidated; in addition to p53-dependent growth suppression, it has strong p53-independent effects in cancer cells (Wanzel et al. 2016 and our unpublished observations). *In silico* screening identified lithocolic acid (LCA), a steroid fatty acid present in bile, as MDMX inhibitor with higher preference over MDM2. It induces apoptosis in HCT116 human cells, predominantly in p53-dependent manner. However, the induction of apoptosis was achieved only at high concentrations of LCA, limiting its use in clinics (Vogel et al., 2012). Previously mentioned stapled peptide SAH-p53-8 also exhibited a high affinity to MDMX, with a 25-fold binding preference for MDMX over MDM2 (Bernal et al., 2010). Another highly selective and dual target stapled peptide, ATSP-7041, displayed a robust p53-dependent tumor growth suppression in xenograft cancer models with MDM2/MDMX overexpression (Chang et al., 2013). Dual D-peptide inhibitor ^D^PMI-α induced a significant reduction in tumor volume in human glioma xenograft model (Liu et al., 2010).

### Dual MDM2/XIAP inhibitors

In addition to controlling p53, MDM2 can bind to several mRNAs via its RING domain, including XIAP mRNA, and regulate their translation. XIAP binds to and inhibits major caspases thus blocking apoptosis. It has been associated with the development of resistance to chemotherapy in several tumor types (Obexer and Ausserlechner, 2014). Upregulation of XIAP in human cancers has been correlated with a poor prognosis (Tamm et al., 2004; Mizutani et al., 2007; Hussain et al., 2017). Therefore, simultaneous inhibition of MDM2 and XIAP could serve as a powerful strategy to target cancer. Dual MDM2/XIAP inhibitors have been identified by high-throughput screening of chemical libraries using a protein–RNA fluorescence polarization assay (Lubing Gu et al., 2016). Treatment of cells with MX1, MX3, or MX69 decreased the expression of both MDM2 and XIAP and induce apoptosis through the activation of caspases 3, 7, and 9. These compounds can also induce apoptosis in p53-deficient cancer cells expressing both MDM2 and XIAP. Importantly, MX69 exhibit low toxicity and anti-tumor activity in xenograft models (Lubing Gu et al., 2016).

### Targeting upstream regulators

p53 post-translational modifications play a very important role in p53 activity and stability and therefore, targeting the enzymes regulating such modifications has high potential for drug development. Deacetylation of p53 by sirtuins SirT1 and SirT2 strongly inhibits its activity (Luo et al., 2001). Sirtuin inhibitors tenovins (Lain et al., 2008) and inauhzin (Zhang et al., 2012) can activate p53 and trigger apoptosis *in vitro* and *in vivo*. SirT2 can be selectively targeted by the structurally related compounds AEM1 and AEM2, inducing p21 and p53 pro-apoptotic transcriptional targets PUMA and NOXA (Hoffmann et al., 2014).

p53 response is known to be triggered by depletion of pyrimidine biosynthesis due to the suppression of the dihydroorotate dehydrogenase enzyme (DHODH) (Khutornenko et al., 2010). DHODH inhibitor HZ05 induces p53 synthesis, promotes apoptosis, and acts in a synergistic manner with nutlin3a, reducing tumor growth *in vivo* (Ladds et al., 2018).

Compounds that prevent the interaction of human papilloma virus (HPV) oncogene E6 with p53, such as leptomycin B (LMB) and RITA, can be applied in HPV-positive cancers where proteasomal degradation of p53 by E6 is critical for the survival of cancer cells. LMB blocks nuclear export by inhibition of the export protein CRM1 (Freedman and Levine, 1998), while RITA binds to p53 N-terminus and promotes conformational change, preventing the binding of E6 (Zhao et al., 2010).

Upstream regulators of MDM2/X are attractive targets for the design of p53-reactivating compounds. For example, it has been shown that upon nucleolar stress, ribosomal proteins (RPL5, RPL11, RPL23) are released from nucleoli and bind to MDM2, triggering p53 activation (Deisenroth and Zhang, 2011). Several non-genotoxic DNA-intercalating compounds and/or RNA PolI/II inhibitors promote the release of ribosomal proteins, leading to p53 activation and cancer cell elimination. Examples of this kind of compounds are cyclin-dependent kinase (CDK) inhibitor roscovitine (David-Pfeuty et al., 2001), actinomycin D (Choong et al., 2009), BMH-21 (Peltonen et al., 2014), and CX5461 (Bywater et al., 2012).

Another class of non-genotoxic small molecules that bind to DNA is curaxins (Gasparian et al., 2011). These compounds simultaneously activate p53 and inhibit NF-κB without causing detectable genotoxicity. The intercalation of curaxins into DNA cause the ‘chromatin trapping’ of the facilitates chromatin transcription (FACT) complex, which in turn leads to phosphorylation of the p53 Ser392 by casein kinase 2 and inhibition of NF-κB-dependent transcription. Importantly, curaxins suppressed tumor growth in different types of human tumor xenografts grown in mice and are currently being tested in clinical trials (Table 1).

**Table 1:** Clinical trials with p53-activating compounds.

	**Compound**	**Phase**	**Type of tumor**	**Combination therapy**	**Status**	**Clinical trial ID**
**MDM2/X inhibitors**	RG7112 RO5045337	I	Hematologic neoplasms		Completed	NCT00623870
		I	Liposarcomas prior to debulking surgery		Completed	NCT01143740
		I	Solid tumors		Completed	NCT01164033
		I	Soft tissue sarcoma	Doxorubicin	Completed	NCT01605526
		I	Acute myelogenous leukemia	Cytarabine	Completed	NCT01635296
		I	Patients participating in previous Roche-sponsored cancer studies		Completed	NCT01677780
		I	Advanced solid tumors		Completed	NCT00559533
	RG7388 RO5503781 Idasanutlin	I	Solid tumors		Recruiting	NCT03362723
		I	Solid tumors		Completed	NCT02828930
		II	Hydroxyurea-resistant/intolerant polycythemia vera		Recruiting	NCT03287245
		I/II	Relapsed/refractory (R/R) follicular lymphoma (FL) and R/R diffuse large B-cell lymphoma (DLBCL)	Obinutuzumab in R/R FL Rituximab in R/R DLBCL	Recruiting	NCT02624986
		I/II	Relapsed multiple myeloma	Ixazomib citrate Dexamethasone	Suspended	NCT02633059
		I	Advanced malignancies except leukemia		Completed	NCT01462175
		I/Ib	Acute myelogenous leukemia	Alone/cytarabine	Completed	NCT01773408
		I	Solid tumors	Posaconazole	Completed	NCT01901172
		I	Polycythemia vera and essential thrombocythemia		Active, not recruiting	NCT02407080
		I/II	R/R multiple myeloma with TP53 (17p) deletion	Ixazomib Dexamethasone	Suspended	NCT02633059
		III	R/R acute myelogenous leukemia	Cytarabine	Recruiting	NCT02545283
		I/II	R/R FL and R/R DLBCL	Obinutuzumab + venetoclax in R/R FL Rituximab + venetoclax in R/R DLBCL	Recruiting	NCT03135262
		I/II	R/R acute myeloid leukemia (AML) patients not eligible for cytotoxic therapy	Venetoclax	Recruiting	NCT02670044
	MI-773 SAR405838	I	Advanced cancer		Completed	NCT01636479
		I	Solid tumors (advanced cancer)	Pimasertib	Completed	NCT01985191
	JnJ-26854165	I	Advanced stage or refractory solid tumors		Completed	NCT00676910
	MK-8242	I	Advanced solid tumors		Terminated	NCT01463696
		I	Acute myelogenous leukemia	Alone/cytarabine	Terminated	NCT01451437
	DS-3032b	I	R/R multiple myeloma		Recruiting	NCT02579824
		I	FLT3-ITD mutant with R/R AML	Quizartinib	Not yet recruiting	NCT03552029
		I	Advanced solid tumors or lymphomas		Recruiting	NCT01877382
		I	Hematological malignancies		Recruiting	NCT02319369
	CGM097	I	Advanced solid tumors with wild-type p53		Active, not recruiting	NCT01760525
	HDM201	Ib/II	Liposarcoma, excluding p53 mutant	LEE011	Active, not recruiting	NCT02343172
		I	Metastatic uveal melanoma	LXS196	Recruiting	NCT02601378
		I	Neuroblastoma with wild-type p53 and without mutations in ALK and RAS-MAPK pathways		Recruiting	NCT02780128
		I	Advanced solid and hematological tumors with wild-type p53		Recruiting	NCT02143635
		AMG232	Ib	Wild-type p53 soft tissue sarcoma	Radiation therapy	Recruiting	NCT03217266
			Ib	R/R or newly-diagnosed AML	Decitabine	Recruiting	NCT03041688
			0/I	Recurrent or newly diagnosed glioblastoma with wild-type p53		Recruiting	NCT03107780
			I	R/R multiple myeloma	Carfilzomib Lenalidomide Dexamethasone	Recruiting	NCT03031730
			Ib/IIa	Metastatic cutaneous melanoma	Trametinib Dabrafenib	Active, not recruiting	NCT02110355
			I	Advanced solid tumors or multiple myeloma		Completed	NCT01723020
			Ib	R/R AML	Alone/trametinib	Completed	NCT02016729
		ALRN-6924	I/IIa	Advanced solid tumors or lymphomas with wild-type p53		Recruiting	NCT02264613
			I/Ib	R/R AML or advanced myelodysplastic syndrome with wild-type p53	Alone/cytarabine	Recruiting	NCT02909972
**Ribosomal protein activators**	Actinomycin D	III	Low-risk gestational trophoblastic neoplasia	Methotrexate	Active, not recruiting Completed	NCT01823315 NCT01535053 NCT00003702
		II	Persistent or recurrent gestational trophoblastic neoplasia		Completed	NCT00003688
		I	Childhood cancers	Vincristine	Completed	NCT00674193
		II	Advanced unresectable melanoma of the extremity	Ipilimumab and melphalan	Completed	NCT01323517
		III	Newly diagnosed low-risk rhabdomyosarcoma	Vincristine Sargramostim Filgrastim Cyclophosphamide Irinotecan w/wo radiotherapy	Active, not recruiting Completed	NCT00075582 NCT00002995 NCT00354835
		III	Previously untreated rhabdomyosarcoma	Cyclophosphamide Vincristine	Completed	NCT00003958
		III	Younger patients who are undergoing surgery for newly diagnosed stage I, stage II, or stage III Wilms’ tumor	Vincristine Doxorubicin	Completed	NCT00352534
		III	Intermediate risk rhabdomyosarcoma	Vincristine Cyclophosphamide Irinotecan w/wo temsirolimus	Recruiting	NCT02567435
		II	Soft tissue sarcoma of the arm or leg that cannot be removed by surgery	Melphalan	Completed	NCT00004250
		III	Choroid plexus tumors	Vincristine Doxorubicin Cisplatin	Suspended	NCT01014767
	Roscovitine	IIb	Non-small cell lung cancer		Terminated	NCT00372073
	Seliciclib	I	Advanced solid tumors	Sapacitabine	Recruiting	NCT00999401
	CYC202	I/II	Solid tumors		Recruiting	NCT02719977
**Curaxins**	CBL0137	I	Hematological malignancies		Recruiting	NCT02931110
		I	Metastatic or unresectable advanced solid neoplasm		Recruiting	NCT01905228

Deubiquitinase USP7 (also known as HAUSP) plays a critical role counteracting p53 and MDM2 degradation, making it an interesting target for the development of inhibitors (Tavana and Gu, 2017). Although USP7 can interact with both MDM2 and p53 through the TRAF-like domain in a mutually exclusive way, MDM2 interaction has much higher affinity. Paradoxically, partial reduction of USP7 levels destabilized endogenous p53, while nearly complete ablation or genetic disruption of USP7 stabilized p53 levels (Cummins et al., 2004; Li et al., 2004). The first characterized USP7 inhibitor was HBX 41108 (Colland et al., 2009). It induces cell cycle arrest and apoptosis in cells harboring wild-type p53, but also in cells with mutant p53, suggesting secondary targets (Reverdy et al., 2012). Treatment with USP7 inhibitors P22077 (Altun et al., 2011), P0591 (Chauhan et al., 2012), and FT671 (Turnbull et al., 2017) displayed a significant reduction of tumor growth. Recently a more potent and selective allosteric USP7 inhibitors have been discovered. These inhibitors induced degradation of MDM2, stabilization of p53, and induction of p21 in several cancer lines (Gavory et al., 2017). USP7 inhibitors GNE-6640 and GNE-6776 were developed using nuclear magnetic resonance-based screening and structure-based design. Both compounds decreased cell proliferation and activated caspases in p53 wild-type cell lines, but also to a less extent in p53-null cells. In addition, the combination of GNE-6640 and GNE-6776 with DNA-damaging agents and PIM kinase inhibitors enhanced USP7 inhibitor efficacy (Kategaya et al., 2017).

### p53-reactivating compounds in clinics

Disrupting MDM2/X–p53 interaction in clinics can be potentially useful in cancers with low frequency of p53 mutations, i.e. hematological malignancies (Tisato et al., 2017). An impressive number of clinical trials of wild-type p53-activating compounds are currently being performed. These molecules are under clinical evaluation for AML, multiple myeloma, and other hematological malignancies (Table 1). Although most of the studies are still in Phase I/II, MDM2 inhibitor RG7388 (idasanutlin) is now undergoing Phase III investigation for relapsed or refractory AML in combination with cytarabine, a DNA synthesis inhibitor. The study is recruiting participants; no results have been published yet. In a previous Phase I/Ib study, a correlation between improved outcomes in AML patients with high levels of the MDM2 protein has been found, thus concluding that MDM2 protein expression could be an useful biomarker to identify patients who might benefit from RG7388-based therapy (Reis et al., 2016). A variety of AML cell lines and primary AML blast cells have been used to identify which factors confer sensitivity to MDM2 antagonist idasanutlin in combination with MEK inhibitor cobimetinib. The study concluded that AML cells with normal karyotype and wild-type status of *TP53* with elevated FLT3 and MDM2 expression are most sensitive to the combined treatment with cobimetinib and idasanutlin (Seipel et al., 2018).

However, not all patients with high MDM2 level respond equally well to MDM2 inhibitors. Recent study has identified a predictive gene signature, which determines sensitivity to MDM2 inhibition by DS-3032b, and validated it in patient-derived tumor xenograft (PDX) models and *ex vivo* in human AML cells. Although this gene signature is still too broad, the attempts are being made to produce a more feasible signature (Ishizawa et al., 2018). A gene expression signature consisting of 13 upregulated p53 target genes predicts sensitivity to another MDM2 inhibitor, NVP-CGM097, in both cell lines and in PDX models (Jeay et al., 2015). The presence of p53 target genes in this signature indicates that at least a partially activated p53 pathway is necessary to confer sensitivity to NVP-CGM097.

The development of biomarkers and companion diagnostics is ongoing; it is crucial for the success of p53-based therapies. Clinical data from patients involved in trials would hopefully pave a way for the discovery of reliable biomarkers for p53-based therapies.

A number of MDM2 inhibitors are being tested in solid tumors with promising preliminary results. Two clinical trials have been conducted and completed for the small molecule MI-773 in patients with advanced solid tumors. The first study was designed to evaluate the safety and the maximum tolerated dose, as well as pharmacokinetics, biomarkers, and biological effects in solid tumors with no other treatment available, as well as lymphomas. Recently published results from the completed study revealed an accepted safety but, although p53 pathway was activated, it displayed a limited activity as a single agent (de Jonge et al., 2017). Therefore, combination therapy might have potential benefits for patients. In a second Phase I study, a combination of MI-773 with MEK inhibitor pimasertib was assessed in eligible patients with solid tumors with wild-type p53 and RAS/RAF mutations. However, the dose required to achieve the beneficial effects of the combination treatment was associated with a significant later toxicity (De Weger et al., 2015). The results of the study using MDM2 inhibitor MK-8242 as a monotherapy in patients with advanced/refractory solid tumors harboring wild-type p53 have been recently reported (Wagner et al., 2017). The Phase II study concludes that MK-8242 activates the p53 pathway with an acceptable safety and tolerability profile at the recommended dose 400 mg twice a day. The observed partial response and prolonged progression-free survival provide an incentive for further study of MDM2 inhibitors in liposarcoma. Other MDM2 inhibitors such as CGM097, AMG232, HDM201, and ALRN-6924 are currently in clinical development for patients with different types of solid tumors with wild-type p53 status (Table 1).

Ribosomal protein activators such as actinomycin D and roscovitine also have been or are being tested in several Phase I, II, or III clinical trials, either as monotherapy or in combination therapy with other drugs in several type of cancers (Table 1).

## p53-based therapies and immune response

To achieve complete tumor eradication, we need to enhance immunogenicity of tumor cells and the anti-tumor immune response along with targeting pathways crucial for the proliferation and survival of cancer cells (Zitvogel et al., 2013). During the last several years, cancer immunotherapy applying checkpoint inhibitors, such as anti-PD-1, anti-PD-L1, CTLA-4 antibodies and others, to boost immune system generated promising clinical data and a lot of excitement (Weber, 2010). Checkpoint inhibitors decrease the chance of *de novo* resistance and increase the overall survival in melanoma patients (Perier-Muzet et al., 2018). Unfortunately, the checkpoint blockage drugs have shown some degree of organ-specific immune-related adverse events (Baxi et al., 2018).

**Figure 2:**
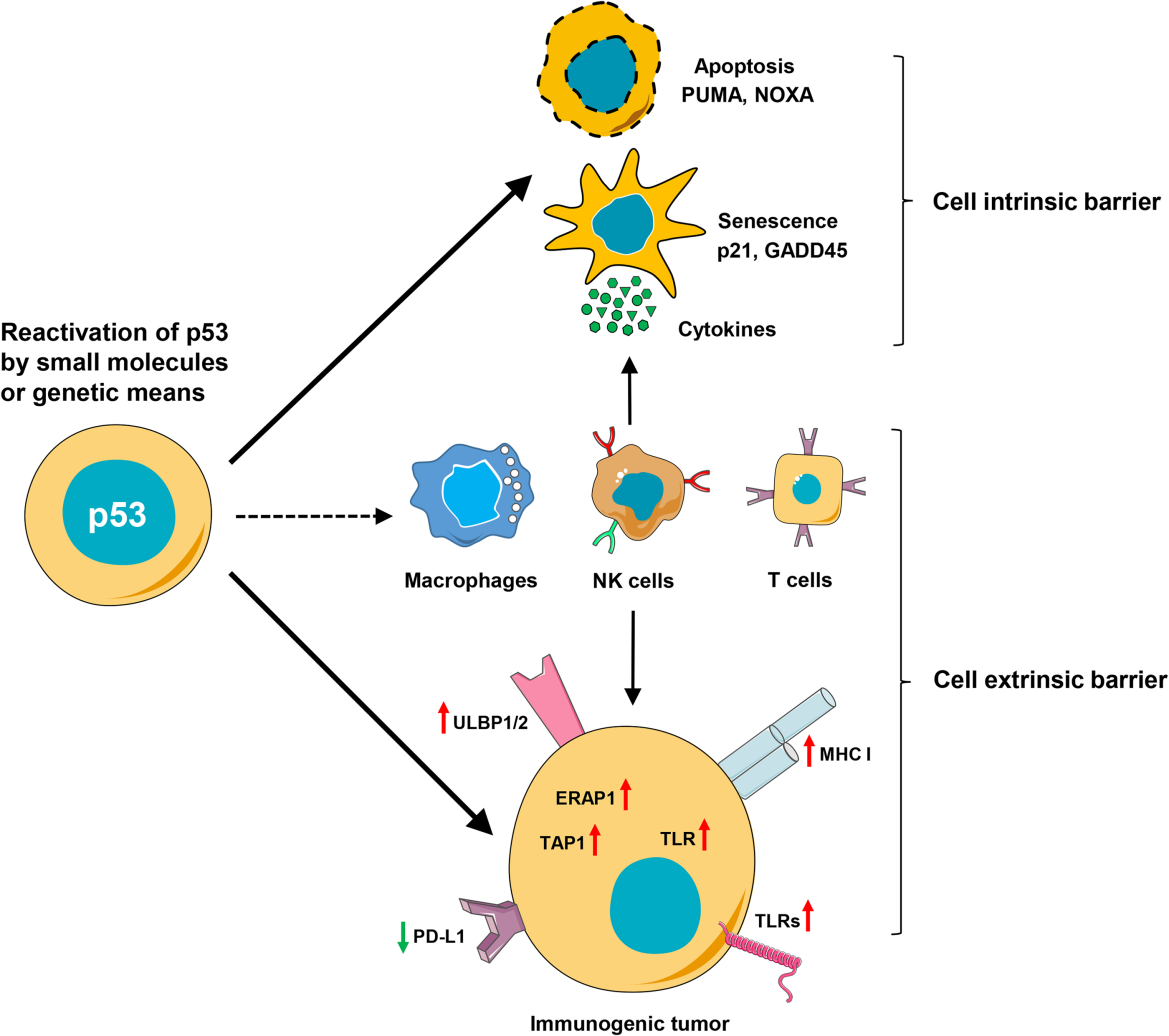
p53 and anti-cancer immune response. Upon activation, p53 upregulates transcription of genes leading to senescence, apoptosis, etc. The senescent cells produce various cytokines (IL6, IL8, CXCL1, etc.), which in turn activate and recruit to tumor site different immune cells, including neutrophils, macrophages, NK cells, and T cells. p53 enhances anti-tumor immunity also by increasing the capacity of tumor cells to present antigens and/or enhancing immune cell infiltration. The major histocompatibility complex (MHC) class I expression for recruitment of cytotoxic T cells, enhancement of NKG2D receptors (ULBP1 and 2) for NK cell activation, Toll-like receptor (TLR) expression for pattern recognition, and inhibition of immune checkpoint molecule PD-L1 via p53 could enhance anti-tumor immunity.

Complementing remarkably different aspects of p53 function, the emerging role of p53 as a regulator of immune surveillance continues to unfold (Li et al., 2012; Cui and Guo, 2016). Recent results suggest that reactivation of p53 can promote both innate and adaptive immunity via multiple molecular pathways and increase the immunogenicity of tumor cells (Figure 2; Tables 2 and 3).

**Table 2:** Regulation of immune-related genes by restored p53 activity in tumor cells.

**Immune-related genes**	**Up- or downregulation**	**Functional outcome**	**Mode of p53 activation**	**References**
ULBP1, ULBP2	Up	Enhanced NK cell-mediated killing of cancer cells	Small molecules (nutlin3a, RITA)	Li et al. (2011); Textor et al. (2011)
APOBEC3 gene family	Up (only A3B down)	Integration of DNA damage and innate immune response	Small molecules (nutlin3a, doxorubicin)	Menendez et al. (2017)
Transporter associated with antigen processing 1 (TAP1), endoplasmic reticulum amino peptidase 1 (ERAP1), MHC class I expression and presentation	Up	Increased expression leads to more efficient antigen presentation	Genetic manipulation, nutlin3a, influenza virus H1N1 and camptothecin, doxorubicin, actinomycin D	Wang et al. (2013); Zhu et al. (1999)
PD-L1	Down	Decreased expression leads to activation of T-cells	Overexpression, nutlin3a	Cortez et al. (2016)
CSF1, MCP1, CXCL1, IL15 (cytokines)	Up	Activated macrophages, NK and neutrophils for tumor clearance	Genetic model	Xue et al. (2007)
TLRs	Up	Increased expression of innate TLRs for pattern recognition	Nutlin3a and p53 overexpression	Shatz et al. (2012)
FAS/APO-1	Up	Increased expression mediates tumor cell killing by T cells	Overexpression of p53	Braun and Iwakuma (2016); Owen-Schaub et al. (1995)

**Table 3:** Effect of p53-reinstatement on immune cells.

**Effector immune cells**	**Functional outcome**	**Mode of activation of p53**	**References**
Neutrophils	Activation to clear senescent cells	Genetic model	Xue et al. (2007)
Macrophages	Activate to clear senescent cells, induce inflammation in tumor cells by induction of IL6 and increase proliferation/activation of M1 macrophages	Genetic model, nutlin3a	Xue et al. (2007); Lowe et al. (2014); Lujambio et al. (2013)
NK cells	Activation of mature NK cells	Mouse models	Collin et al. (2017)
T cells	Inhibit proliferation, while activate of T cells	Nutlin3a, Trp53 KO mice	Madapura et al. (2016); Watanabe et al. (2014)
B cells	B cells differentiation	Genetic models	Molchadsky et al. (2010); Slatter et al. (2010)

Tumor regression by genetically reinstated p53 is associated with the induction of senescence and tumor clearance by macrophages and immune cells in mouse model of liver carcinoma (Xue et al., 2007), via secretion of chemokines, such as CCL2, for the recruitment of NK cells (Iannello et al., 2013). Moreover, factors, secreted by p53-expressing senescent cells, screw macrophage polarization towards tumor-inhibiting M1 state (Lujambio et al., 2013). These findings have been further confirmed in another mouse model, demonstrating that p53 reactivated by nutlin3a suppressed M2 phenotype of macrophages via transcriptional repression of c-Myc (Li et al., 2015).

We and others have found that the treatment of cancer cells with different p53-reactivating compounds induce the expression of ULBP1/2 ligands of the NKG2D receptor, which enhanced NK cell-mediated tumor cell killing (Li et al., 2011; Textor et al., 2011).

Another intriguing aspect of the p53 tumor suppression is the control of adaptive immunity. The direct binding of p53 to the promoter of gene encoding IL-12 facilitates dendritic cell function and promotes adaptive immunity (Slatter et al., 2016). Notably, p53 regulates the expression of several genes important for tumor cell recognition by the cytotoxic T-lymphocytes (CTLs), including the repression of PD-L1 via upregulation of miR-34 (Cortez et al., 2016). Furthermore, p53 unleashes CTL response by inducing the expression of several other genes, such as MHC class I, TAP1, ERAP1, and apoptosis signal receptor Fas/APO1 (Wang et al. 2013; Zhu et al. 1999; Table 2).

Modelling *in vivo* response upon local reactivation of p53 by intra-tumor injection of nutlin3a demonstrated elimination of tumor cells via two non-redundant p53-dependent processes: reversal of immunosuppression in tumor microenvironment and induction of immunogenic cell death, leading to the activation of dendritic cells, macrophages, and CD8^+^ T cells and resulting in regression of tumors distal to the nutlin3a injection site (Guo et al., 2017).

Taken together, these studies provide a compelling evidence that targeting p53 inhibitors can augment therapeutic benefits of p53-mediated tumor cell killing via engagement of both innate and adaptive anti-tumor immune responses to achieve durable and systemic tumor eradication. Although we still have much to learn about the effects of p53 on immune response, reactivation of p53 represents a fascinating strategy to reverse immunosuppression and boost anti-tumor immunity.

## Challenges and limitations associated with pharmacological reactivation of wild-type p53

One of the main concerns for the therapeutic use of wild-type p53-reactivating compounds is the toxicity for normal cells. In normal fibroblasts and epithelial cells, p53 reactivation has been reported to cause either irreversible or reversible cell cycle arrest, but not apoptosis (Efeyan et al., 2007; Shangary et al., 2008; Korotchkina et al., 2009), which led to the conclusion that p53 reactivation is harmless for normal tissues. Reconstitution of p53 in mice does not result in growth suppression in tissues (Christophorou et al., 2005). However, in the MDM2-null background, sudden reinstatement of p53 in adult mice caused a rapid tissue destruction and death of mice (Ringshausen et al., 2006). This later study underlined the risk of complete MDM2 inhibition in normal tissues. However, the pharmacological inhibition of MDM2 is radically different from the complete deletion of the gene; first of all, because it will never completely inhibit the continuously produced protein, expressed even at higher level due to p53 activation. Experimental evaluation of toxicity of MDM2 inhibitors obtained in mouse models suggests that tumor-suppressing doses of nutlin3a, RITA, MI-219, and stapled peptide ATSP-7041 do not cause weight loss and are well tolerated (Chang et al., 2013). However, the only reliable answers we can get regarding the toxicity of MDM2 inhibitors will be coming from clinical trials.

Phase I studies of RG7112 in patients with liposarcoma and leukemia showed severe hematological toxicity (febrile neutropenia and thrombocytopenia) as the most common adverse effect (Ray-Coquard et al., 2012; Andreeff et al., 2016). The data from ongoing clinical trials will tell us more about the applicability and limitations of wild-type p53-reactivating compounds.

Besides the effects of p53 reactivation in normal cells, another concern of targeting negative regulators of p53 is the inhibition of their cellular functions other than p53 regulation. Both MDM2 and MDMX have several p53-independent functions, including gene expression regulation and chromatin modification (Biderman et al., 2012; Wienken et al., 2017), DNA repair (Eischen, 2017), DNA replication (Frum et al., 2014), and mitochondrial dynamics (Arena et al., 2018). To note, Phase I study of MDM2 inhibitors RG7112 showed a response in AML carrying p53 mutations (Andreeff et al., 2016). Albeit this opens a window for potential therapeutic applications by directly targeting MDM2/MDMX oncogenic functions regardless of p53 mutation status, the p53-independent effects and potential harmful effects of MDM2 inhibition in normal cells and its clinical relevance are not yet completely understood.

As every targeted therapy, wild-type p53 reactivation-based therapy will be efficient only in a subset of patients. Patients should be stratified according to the alterations in the pathways regulating p53, such as MDM2 gene amplification, which occurs in ~7% of tumors (Momand et al. 1998), deletion or inactivation of MDM2 negative regulators such as p14^ARF^, amplification or overexpression of MDM2 positive regulators HAUSP, Wip1, and others (Zhang et al., 1998; Cummins et al., 2004; Lu et al., 2007). However, wild-type p53 can be inactivated by a broader range of mechanisms (Wasylishen and Lozano, 2016). For example, HPV oncoproteins E6 directly binds to p53 and induces its degradation (Scheffner et al., 1990). Moreover, there is a subset of tumors where wild-type p53 is inactive without any alteration in the known MDM2–p53 pathway, for example in renal cell carcinoma (Gurova et al., 2004). Moreover, the response of different cell lines to nutlin3a is variable, ranging from cell cycle arrest to apoptosis (Tovar et al., 2006; Duan et al., 2018). Extensive genome-wide studies have not been able yet to identify exact molecular mechanisms, which dictate the choice between the different biological responses induced by p53 (Allen et al., 2014). It is therefore imperative to identify reliable biomarkers for wild-type p53 reactivation strategies.

Therapeutic response to p53 reactivation by small molecules, as every precision medicine, is limited both by the plasticity of the tumor and by the intra-tumor heterogeneity. These lead to the selection of pre-existing resistant cells or *de novo* emergence of mutations allowing to avoid the effects of therapy (Tannock and Hickman, 2016). By definition, treatment with MDM2 inhibitors confer a strong selection pressure for p53 inactivation. Therefore, considering the genomic instability of cancer cells, the emergence of mutations in the DNA binding domain of p53 upon prolonged treatment *in vitro* with nutlin3a is not surprising (Aziz et al., 2011; Michaelis et al., 2011). This has been confirmed in a clinical context, during Phase I clinical studies with MDM2 inhibitor MI-773 in patients with liposarcoma (Jung et al., 2016). *TP53* mutation burden increased over time during the treatment and was associated with resistance to MDM2 inhibition, leading to a very modest clinical effect. While the selection pressure for p53-mutated cancer cells leads to resistance (and potentially to an increased aggressiveness due to mutant p53 oncogenic gain of function), the possible selection of p53 somatic mutations in normal cells such as hematopoietic progenitors may lead to the development of new cancers and therefore should be carefully investigated. Systematic search for the mechanisms of resistance to MDM2 inhibitors using piggyBac transposon insertional mutagenesis in spontaneous tumors in p19ARE^−/−^ mice revealed several mechanisms of resistance. More than half of tumors acquired inactivating mutations in p53 (54%), while others obtained the gain-of-function alterations resulting in high expression of anti-apoptotic protein Bcl-xL, MDMX, and ΔNTrp63 or ΔNTrp73, which confer a dominant-negative effect on p53 (Chapeau et al., 2017).

## Combination therapy

Although MDM2 inhibitors have shown therapeutic benefits in preclinical studies and in several clinical trials as monotherapy, wild-type p53 reactivation will require combination therapies for efficient clinical use. Since cancer cells can evolve in response to therapy, we need to design drug combinations that prevent the development of acquired resistance, for example, mutations in p53. Therefore, it could be an attractive strategy to combine Mdm2 inhibitors with compounds with specificity toward p53 mutant cells, such as PRIMA1^MET^/APR-246 (Bykov et al., 2002). Recent studies *in vitro* suggest that such combinations are quite efficient (Izetti et al., 2014).

High-throughput approaches could be very useful to identify rational combinations of drugs, which synergize with MDM2 inhibitors. For example, RNA interference screens identified several pathways, whose inhibition promotes the pro-apoptotic effect upon p53 reactivation, including MAP kinase and sphingosine kinase pathway (Cheok and Lane, 2012). ATM and MET kinases are synthetic lethal in combination with non-genotoxic activation of p53 (Sullivan et al., 2012). Further, induction of reactive oxygen species via inhibition of thioredoxin reductase TrxR1 (Shi et al., 2014) or blocking CDKs (Cheok et al., 2007) or Aurora kinase (Cheok et al., 2010) is sufficient to elicit cell death upon co-treatment with nutlin3a. Interestingly, inhibition of autophagy also facilitates apoptotic response upon nutlin3a treatment (Sullivan et al., 2015). Concomitant inhibition of MDM2 and BCL-xL or Bcl-2 demonstrated significant synergy in p53 wild-type cell lines *in vitro* (Carter et al., 2015; Chapeau et al., 2017). Another therapeutic option could be provided by a combination of RG7112 with TNF-related apoptosis inducing ligand (TRAIL) agonist rhTRAIL (Urso et al., 2017).

## Concluding remarks

A number of ongoing clinical trials are using p53-reactivating compounds in combination with different chemotherapeutic drugs, so we might get some clues for patient stratification from these clinical studies. In order to decrease systemic toxicity, it would be essential to find out which targeted therapies (which presumably display limited side effects) will be beneficial to combine with p53-reactivating drugs. However, synergistic or synthetic lethality drug interactions remain a largely unexplored area*.* Therefore, there is an urgent medical need to apply systems biology approaches to rationally design and develop combinations of p53-reactivating compounds with targeted drugs. More systematic validation studies using not only established cancer cell lines, but also extensive collections of molecularly characterized PDX models and patient-derived tumor organoids are required to identify such combinations from a growing list of targeted therapies.
